# Successful treatment of neonatal atrial flutter by synchronized cardioversion: case report and literature review

**DOI:** 10.1186/s12887-020-02259-7

**Published:** 2020-08-05

**Authors:** Monika Wójtowicz-Marzec, Barbara Wysokińska, Maria Respondek-Liberska

**Affiliations:** 1 Department of Obstetrics and Pathology of Pregnancy, Medical University of Lublin, Staszica 16, 20-081 Lublin, Poland; 2 Department of Paediatric Cardiology, Medical University of Lublin, Prof. A. Gębali 6, 20-093 Lublin, Poland; 3 Department of Prenatal Cardiology, Department for Fetal Malformations Diagnoses & Prevention, Medical University of Lodz, Rzgowska 281/289, 93-338 Łódź, Poland

**Keywords:** atrial flutter, tachycardia, fetal, neonatal, arrhythmic drugs, CTG tracing

## Abstract

**Background:**

Atrial flutter (AFL) is a supraventricular tachyarrhythmia. In the ECG tracing, it is marked by a fast, irregular atrial activity of 280–500 beats per minute. AFL is known to be a rare and also life-threatening rhythm disorder both at the fetus and neonatal period. AFL may result in circulatory failure, and in a more severe form, it may lead to a non-immune fetal hydrops. However, with early prenatal diagnosis and proper treatment, the majority of AFL cases show a good prognosis.

**Case presentation:**

We report a case of a neonate who was born at 34 weeks of gestational age by C-section because of risk for birth asphyxia, based on abnormal CTG tracing, which had no characteristic rhythms for fetal decelerations. A third day his heart rate was 220/bpm. ECG has shown supraventricular tachycardia with narrow QRS. The administration of adenosine resulted in the obvious appearance of “sawtooth wave” typical for AFL. Arrhythmia was resistant to the therapy of amiodaron. Then cardioversion was performed and the rhythm converted to normal.

**Conclusions:**

As neonatal AFL might be resistant to conventional pharmacotherapy, one needs to remember about the possibility of electrical cardioversion in the pediatric cardiology referral center. Moreover, CTG monitoring is of limited use because it does not record fetal heart rhythms > 200/min and echocardiography at the reference center is practically the only method to monitor the condition of the fetus with abnormal rapid heart rhythm.

## Introduction

Atrial flutter (AFL) is a rare type of an arrhythmia encountered in children, in the ECG tracing it is marked by a fast, irregular atrial activity 280–500 beats per minute. This arrhythmia is caused by the re-entry circuits limited to the right atrium. It mainly concerns children suffering from congenital heart defects such as: transposition of the great arteries, complex cyanotic heart defect, atrial septal defect, Ebstein’s anomaly, pulmonary stenosis, tricuspid valve diseases. Atrial flutter may develop post-operatively especially, after the cardio-surgery performed within atria: interatrial transposition of the great arteries using the Mustard or Senning procedure. It is also often observed after the Fontan procedure and repair of tetralogy of Fallot [1]. It is worth to mention that this type of atrial flutter is atypical and it mostly depends on a cavotricuspid isthmus.

Atrial flutter may also develop in patients with normal heart anatomy, mainly in newborns or in fetuses.

Then the atrial flutter may result in circulatory failure, and in a more severe form, it may lead to a non-immune fetal hydrops. Perinatal atrial flutter is approximately 9% mortality [2].

The newborns’ ECG tracing shows an atrial flutter wave (so-called “sawtooth”), which is seen in leads II, III, aVF, V1. Clinical features depend on the frequency of the ventricular rate. 1:1 atrioventricular conduction concerns the patients with a coexisting accessory pathway as it predisposes to ventricular fibrillation. However, because of an accompanying atrioventricular block, the ventricular rhythm is usually slower than the atrial rhythm. Atrial Flutter (AFL) is a rare but potentially lethal arrhythmia. Therefore, it is essential to know how to treat it.

## Case presentation

It was a second pregnancy of 34-year-old women with obesity and primary hypertension. High blood pressure of maximum values 170/100 mmHg was treated with methyldopa in increasing doses, trimetazidinie, dihydrochloride and low dosage acetylic acid. Ultrasound exam at the 13th week of gestation was normal with NT 2,2 mm, and at 20 weeks of gestation there was reported normal fetal biometry and normal heart anatomy. At the age of 34th week of pregnancy, woman was admitted to hospital because of high blood pressure and abnormal Doppler flow in fetus. No echocardiography exam was available. Antenatal corticosteroids were given once three days before birth with magnesium sulfate for fetal neuroprotection and lung development stimulation.

**Fig. 1:**
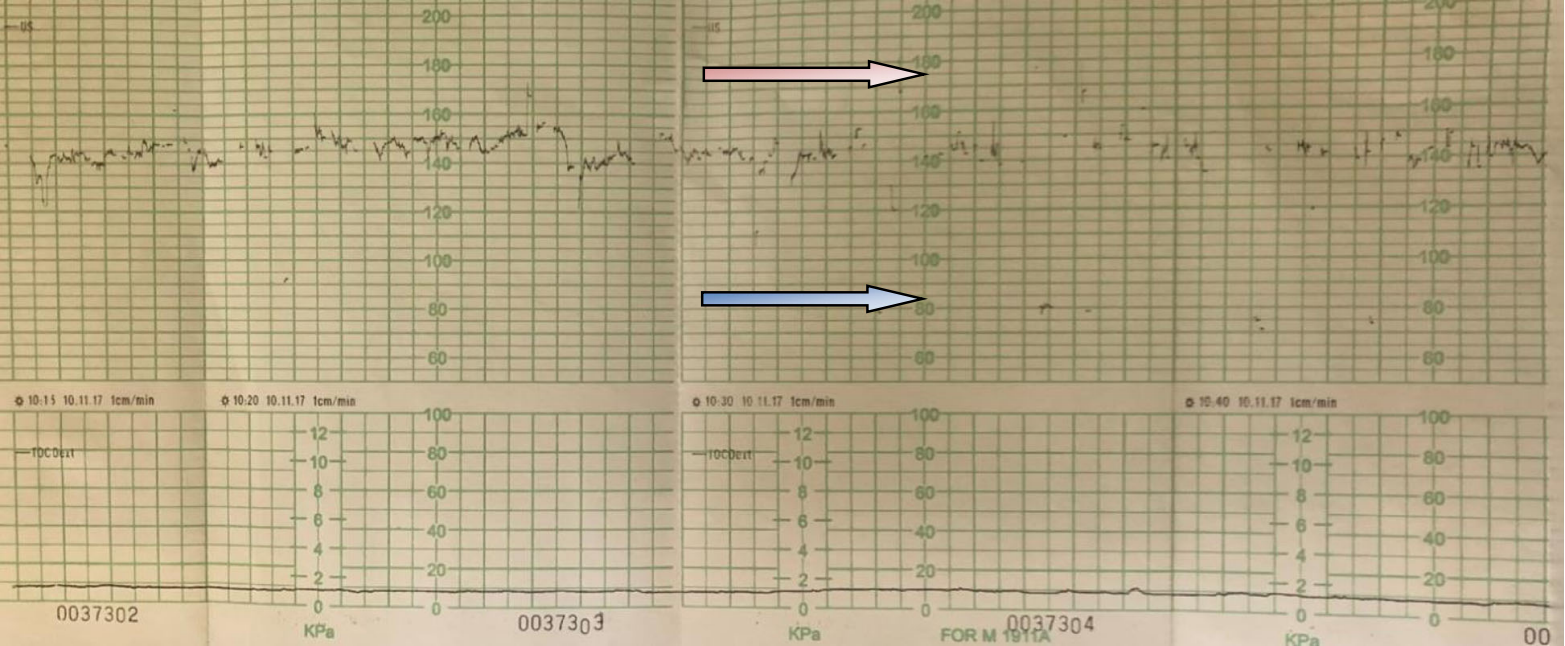
Abnormal CTG tracing shows inserts of slow rhythms (blue arrow) that do not correspond to characteristic for life-threatening fetal decelerations. There are dot inserts of fast rhythms (red arrow), which are not typical for fetal tachycardia. This type of CTG trace is an indication for ultrasound examination. Unfortunately no echocardiography exam was available

In a district hospital, a male neonate was born at 34 weeks of gestational age by C-section because of increased risk for birth asphyxia, based on abnormal cardiotocography (CTG) tracing (Fig. 1).

**Fig. 2:**
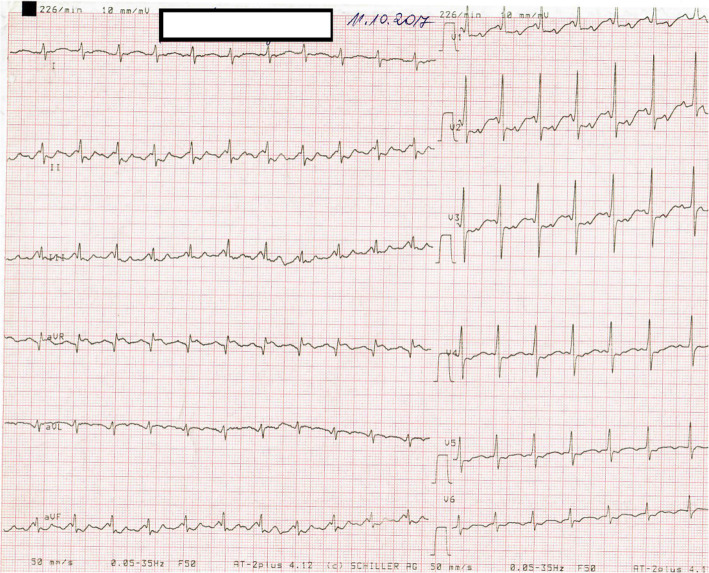
Atrial flutter. The flutter rate is 500 beats/min with 2:1 conduction giving a ventricular rate of 215–220 beats/min (50 mm/s)

**Fig. 3:**
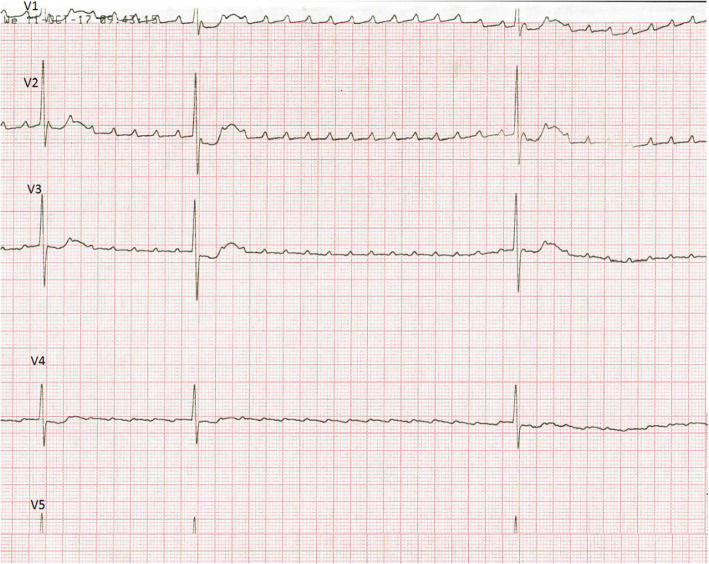
Administration of adenosine exposed the etiology of the tachycardia by exhibiting ‘saw tooth’ flutter waves (50 mm/s)

The preterm boy had birth weight 2600 g and had head circumference and length in normal values range of 50–90 centiles. Due to respiratory distress syndrome, nasal Duo positive airway pressure was performed for the first two days of postnatal life. The chest x-ray excluded pneumonitis, the heart size was in normal values. Laboratory findings show increasing C-reactive protein concentrations in the following days. A third day at the physical exam, his heart rate was 220/bpm. ECG has shown the supraventricular tachycardia with a narrow QRS atrial rate (AR) was equal to ventricular (VR) and was 220/min. Adenosine was administered twice- first dose 0,15 mg/kg, second dose 0,25 mg/kg. But arrhythmia remained resistant to these therapies. However, ECG detected for a short time atrial tachycardia (atrial rate 420/min, ventricular rate 45-65-70/min). Therefore, amiodarone therapy was started- 5 mg/kg. Echocardiography revealed patent foramen ovale, tricuspid valve regurgitation with a 29 mmHg gradient and mild mitral regurgitation. The ejection fraction (EF) was 62%. Because of unsuccessful treatment, the newborn was transferred from the district hospital to hospital with a Paediatric Cardiology Department on day 3. Next ECG demonstrated supraventricular tachycardia with narrow QRS (220/min) (Fig. 2). The administration of adenosine (0,1 mg/kg) resulted in the obvious appearance of “sawtooth wave” typical for AFL (Fig. 3). After a while, ECG demonstrated supraventricular tachycardia (SVT), exactly 3:1 atrioventricular conduction AFL (atrial rate 500/bpm, ventricular rate 250/bpm). Due to the recurrence of AFL, cardioversion was performed with 1 J/kg and the rhythm converted to normal. Amiodarone therapy with a dosage of 15 mg/kg/day was started as a prophylaxis against recurrent arrhythmia attacks. After 24 h without the AFL attack, intravenous amiodarone therapy was replaced with oral treatment. SVT did not occur and the infant was discharged on the 23rd day of life in a good general state with amiodarone oral therapy. AFL did not repeat in the 1-year follow up and corrected QT (QTc), Holter ECG and echocardiography (ECHO) were all found to be normal. Therefore, therapy was stopped.

## Discussion and Conclusions

Atrial flutter is characterized by saw-tooth flutter waves with an atrial rate of up to 500 beats/min and is frequently associated with 2:1 atrioventricular (AV) conduction. The mechanism of AFL is macro reentry within the atrial wall. AV block does not terminate AFL because the AV node is not involved in the reentrant circuit. For this reason, adenosine cannot terminate AFL but unmasks the flutter wave by causing AV block.

Atrial flutter accounts for near one-third of fetal tachyarrhythmias. AFL is observed only in the third trimester, which is probably related to the large atrial size achieved at 27–30 weeks of gestation, with high vulnerability to atrial extrasystoles. Accessory AV pathways and reentrant SVT are a common association. This fetal arrhythmia may be associated with myocarditis, positive SSA/SSB autoantibody or congenital heart disease such as Ebstein anomaly. Fetal AFL can cause hydrops fetalis or fetal heart failure.

In fetuses with AFL, sotalol is recommended in case of hydrops. Sotalol has been effective in converting 50–80% of fetuses with atrial flutter without mortality. Digoxin or amiodarone may be considered as the second choice. Procainamide is contraindicated [Table 1] [2–7].

**Table 1:** Arrhythmic drugs recommend in fetal atrial flutter based on statement AHA [3]

**Drug**	**Therapeutic maternal dose range**	**Therapeutic level and effect**	**Toxicity**
Digoxin	LD: 1200–1500 µg/24 h IV, divided every 8 h MD: 375–750 µg/d divided every 8 to 12 h PO (Fetal intramuscular dose: 88 µg/kg q12 h, repeat 2 times)	0.7–2.0 ng/mL Maternal Nausea, fatigue, loss of appetite, sinus bradycardia, first-degree AV block, rare nocturnal Wenckebach AV block	Nausea/vomiting +++, sinus bradyarrhythmia or AV block +++, proarrhythmia Fetal intramuscular: sciatic nerve injury or skin laceration from injection
Sotalol	160–480 mg/d divided every 8 to 12 h PO	Levels not monitored Bradycardia, first-degree AV block, P and QRS widening, QTc ≤ 0.48 s	Nausea/vomiting, dizziness, QTc ≥ 0.48 s, fatigue, BBB, maternal/fetal proarrhythmia
Amiodarone	LD: 1800–2400 mg/d divided every 6 h for 48 h PO; lower (800–1200 mg PO) if prior drug therapy MD: 200–600 mg/d PO Consider discontinuation of drug and transition to another agent once rhythm is converted or hydrops has resolved.	0.7–2.8 µg/mL Maternal/fetal sinus bradycardia, decreased appetite, first-degree AV block, P and QRS widening, QTc ≤ 0.48 s	Nausea/vomiting ++, thyroid dysfunction ++, photosensitivity rash, thrombocytopenia, BBB, QTc ≥ 0.48 s, maternal/fetal proarrhythmia, fetal torsades with LQTS, fetal goiter, neurodevelopmental concerns

Proarrhythmia means worsening of an arrhythmia as the result of treatment. AV indicates atrioventricular block; *BBB* bundle-branch block; *CNS* central nervous system; *ECG* electrocardiogram; *IV* intravenously; *LD* loading dose; *LQTS* long QT syndrome; *MD* maintenance dose; *PO* orally; *VT* ventricular tachyarrhythmia; and +++, very common; ++, common; and +, occasional.

Preparing a newborn baby with atrial flutter in the prenatal period is particularly important. Sinus node suppression may occur, although rarely, from in utero drug therapy. In such a condition, it is important to be prepared with backup external pacing. After delivery, medical treatment must be reassessed given that the arrhythmia may not recur.

Neonatal sinus rhythm can be restored by electrical cardioversion, transoesophageal pacing or antiarrhythmic drugs. The recommended treatment for a newborn, both stable and unstable, is either synchronized electrical cardioversion or transoesophageal atrial overdrive. In a stable newborn antiarrhythmic drugs can be tried. However, it takes some time to restore sinus rhythm. The recommended drugs are digoxin with the addition of flecainide or amiodarone in case of no therapeutic effect [1]. There are available studies that reviewed a group of infants with AFL [8, 9]. According to these reports, direct current cardioversion appears to be the most effective treatment of AFL.

In our case, the mother was obese and the preterm neonate birth weight was near the 90th centile. Trying to connect these clinical findings, we found the original article [10] suggesting more likely that fetuses and neonates with atrial flutter or ectopic atrial tachycardia, are born to diabetic mothers than in the general population. The authors suggest that ectopic atrial tachycardia may be caused by cardiac diastolic dysfunction and atrial stretch in utero. What is more, Linda et al. [11] report a positive association between maternal body mass index during pregnancy and maternal height and previously atrial fibrillation in offspring. There is a suggestion that the prevention of maternal obesity might reduce later atrial fibrillation in offspring. No studies are describing an association between maternal obesity and atrial flutter in adulthood.

We can state that that AF is a serious and life-threatening rhythm disorder both at the fetus and neonatal period. Specifically, when it causes hydrops, it is associated with fetal death. CTG monitoring is of limited use because it does not record fetal heart rhythms > 200/min and echocardiography at the reference center is practically the only method to monitor the condition of the fetus with abnormal rapid heart rhythm [12]. Treatment of fetal AF should be carried out at a reference center. The management of fetal tachycardia depends on the effects of cooperation with the medical team: obstetrician, fetal cardiologist, internist cardiologist. Both the fetus and the pregnant are monitored during treatment.

As neonatal atrial flutter might be resistant of conventional pharmacotherapy, one needs to remember about the possibility for electrical cardioversion in the pediatric cardiology referral center. Within a few months after delivery, pharmacological prophylaxis is used to prevent atrial flutter. An ultrasound assessment of the brain during the first months of life is also recommended to exclude hypoxic changes in the brain [13]. Once the fetus and newborn with AF survive, its future is bright, and prophylaxis beyond the infant period is unnecessary.

## Data Availability

The datasets analyzed during the current study are available from the corresponding author on reasonable request.

## Abbreviations

AFL: Atrial flutter
CTG: Cardiotocography
AR: Atrial rate
VR: Ventricular rate
SVT: Supraventricular tachycardia
ECHO: Echocardiography
AV: Atrioventricular

## Constent for publication

Written informed consent was obtained from the parents for publication of this case report and any accompanying images.
